# Coaching to Support Mental Health Apps: Exploratory Narrative Review

**DOI:** 10.2196/28301

**Published:** 2022-03-08

**Authors:** Ashley Meyer, Hannah Wisniewski, John Torous

**Affiliations:** 1 Division of Digital Psychiatry Department of Psychiatry Beth Israel Deaconess Medical Center Boston, MA United States

**Keywords:** smartphone, apps, mental health, coaching, engagement

## Abstract

**Background:**

The therapeutic alliance is crucial for the success of face-to-face therapies. Little is known about how coaching functions and fosters the therapeutic alliance in asynchronous treatment modalities such as smartphone apps.

**Objective:**

The aim of this paper was to assess how coaching functions and fosters the therapeutic alliance in asynchronous treatment modalities.

**Methods:**

We conducted a selected review to gather preliminary data about the role of coaching in mobile technology use for mental health care. We identified 26 trials using a 2019 review by Tønning et al and a 2021 scoping review by Tokgöz et al to assess how coaching is currently being used across different studies.

**Results:**

Our results showed a high level of heterogeneity as studies used varying types of coaching methods but provided little information about coaching protocols and training. Coaching was feasible by clinicians and nonclinicians, scheduled and on demand, and across all technologies ranging from phone calls to social media.

**Conclusions:**

Further research is required to better understand the effects of coaching in mobile mental health treatments, but examples offered from reviewed papers suggest several options to implement coaching today. Coaching based on replicable protocols that are verifiable for fidelity will enable the scaling of this model and a better exploration of the digital therapeutic alliance.

## Introduction

Smartphones and other mobile technologies are increasingly used in mental health care. The COVID-19 pandemic has highlighted the need for mobile treatments in providing access to and augmenting mental health care. However, fundamental questions remain around app engagement and efficacy as do concerns about technology use in the contact of coaching support and the therapeutic alliance. The therapeutic alliance, which is described as the alliance between a clinician and a patient [1], is considered crucial to the success of face-to-face therapies and associated with successful outcomes [2]. Numerous meta-analyses have confirmed the central role of the therapeutic alliance in driving both engagement and efficacy across both face-to-face care and even telehealth video visits. However, less is known about how this alliance functions in asynchronous treatment modalities such as smartphone apps. In both research studies as well as commercial apps, a rise in coaching to support engagement may be conceptualized in the context of adding an element of the therapeutic alliance into digital care. Preliminary research suggests that coaching is feasible and acceptable, with both clinicians and clients identifying benefits to this additional support, such as increased motivation and guidance as well as a new way to focus on clinical work [3]. Yet, little is known about how this app coaching is delivered or impacts outcomes.

This lack of knowledge on the therapeutic alliance and coaching around smartphone apps is in part related to a lack of consensus on coaching methods and training. Such a lack of protocols or training manuals has immediate consequences as well-trained clinicians are more prepared to foster the therapeutic alliance and support technological difficulties [4]. At the beginning of the COVID-19 pandemic, 21% of community health centers across the United States lacked training for telehealth [5]. Training for mobile apps is nascent, and evidence can often only be found in the research literature. For example, one study in Australia found that clinician training on mobile technologies and protocols for consistent messaging with the patient was necessary to increase engagement and knowledge of the apps [6]. A recent report from Kaiser Permanente explained that teaching clinical staff how to use and interact with apps in care settings was critical for implementation success [7]. With COVID-19, grassroots efforts to train medical students to support patients around app use have also emerged [8]. The need for proper training and new knowledge around engagement and alliance to support app use is thus an important new facet of care toward offering accessible mental health care in an increasingly digital world. The term “training” is used throughout this work and defined based on the above references as one or more of the following: the receipt of coaching protocols or manuals by the coach, instruction for the coach on how to conduct telehealth sessions, how to use and interact with mobile apps, and how to support patients around app use.

Several solutions have been proposed already. Many have examined a more traditional “coaching” model in which a member of the care team maintains contact with patients between visits to foster engagement with the mobile technology. This contact may be through the app, text messages, or even phone calls. A more engaged approach is conceptualized around the concept of a “digital navigator,” which seeks to integrate into more aspects of care with the goal of supporting both the patient and clinician [9]. Ben-Zeev et al [10] and Noel et al [11] have proposed related roles called clinical technology specialists or technology specialists, respectively. Each of these positions seeks to provide technology recommendations and support user engagement with digital technologies.

A related method for maintaining alliance with patients using digital technology uses a “social” model of coaching. This model focuses on interactions between peers as opposed to a member of the clinical or study team. Although this model of coaching appears less used, several studies have incorporated this method. McEnery et al [12] evaluated the feasibility of an online intervention, EMBRACE, where participants maintained contact with clinical moderators, a more traditional coach, as well as peer-to-peer moderators, who were young individuals with lived mental health experience who encouraged participant engagement and provided support. Further, Alvarez-Jimenez et al [13] assessed the feasibility and acceptability of the enhanced moderated online social therapy (MOST+), which allowed participants to interact with other participants using the platform, as well as peer moderators who facilitated engagement. However, neither of these protocols directly assessed the effect of moderation on outcomes, and, of note, neither involved smartphone apps.

To further understand the current knowledge of coaching effects, we conducted an investigation to gather preliminary data on coaching and to understand its effect on engagement and outcomes. We hypothesized that there would be substantial variability and little consensus on coaching protocols, inconsistent reporting measures, and a lack of protocols that directly assess coaching effects. However, understanding how coaching is reported and broad trends in its outcomes is useful for new efforts to align new research and implementation efforts with prior work. In turn, understanding and identifying best practices to facilitate coaching and support the digital therapeutic alliance is crucial as remote psychotherapy increases in popularity and necessity.

## Methods

We conducted a selected review to gather preliminary data about the role of coaching in mobile technology use. Realizing there is no simple means to identify relevant papers as all will have some degree of coaching support, often unreported in research assistant help, we opted for an exploratory sampling approach. We chose to use a prior review of randomized controlled trials on smartphone-based treatment in psychiatry as well as a recent scoping review on digital health interventions for depression (featuring 6 smartphone-based interventions) as the samples for assessing how coaching is offered across different apps and studies. We did not attempt to conduct a qualitative analysis as we expected outcomes to be heterogeneous and diverse given the state of the literature.

We identified the trials using the 2019 review by Tønning et al [14] on the methodological challenges of randomized controlled trials on smartphone-based treatment in psychiatry and the 2021 scoping review by Tokgöz et al [15] on digital health interventions for depression. We selected these papers as they offered a recent and comprehensive sample of studies from which we could explore coaching. Each trial featured in this review was read and coded by the authors for method of contact with the patient, training coaches received, on demand vs scheduled interactions, clinical vs nonclinical interactions, evidence of dose effect, and social vs coaching model. Studies were excluded if the participants had no interaction while using the mobile technology.

After coding of each trial was completed, the trials were sorted into the following 4 categories based on the frequency (scheduled vs on demand) and nature of the coaching (nonclinical vs clinical): scheduled or clinical; scheduled or nonclinical; on demand or clinical; and on demand or nonclinical. Scheduled coaching included coaching delivered on a set time frame, such as once per week or per month, or after the completion of a certain assessment. On-demand coaching was delivered on an irregular schedule based on the needs of the study participant or clinician, such as clinician responses to high participant assessment score or a participant contacting study team for questions; however, we did not include on-demand crisis intervention in this category and will not be reviewing such interventions within this work. Clinical coaching focused on the participant’s symptomatology, whereas nonclinical coaching focused on technology or study protocol questions. Some trials that included a variety of methods for interaction were coded into multiple categories. We then assigned a hierarchy to the codes as follows from highest priority to lowest priority: (1) on demand or clinical; (2) on demand or nonclinical; (3) scheduled or clinical; and (4) scheduled or nonclinical. Dose effect was defined as a reported association between the time or intensity of coaching for the participants and a primary study outcome. While the digital therapeutic alliance is of critical interest, it is not yet possible to code given that the means to assess it are nascent, as discussed later in this paper.

## Results

A total of 32 trials were reviewed as featured in the 2019 review by Tønning et al and 2021 scoping review by Tokgöz et al; 6 of these studies did not involve reported coaching interaction while the participants were using the smartphone technology and were excluded from review by the study team [16-21]. Therefore, a total of 26 trials were included [22-47]. A summary of the results can be seen in Multimedia Appendix 1 and Table 1.

As seen in Table 1, there was high variability around coaching across each of the 26 studies in the type of coaching delivered and coach training. The majority of studies included a scheduled coaching component (14 scheduled or clinical) [22,23,25,26,28,29,31,33,36,38,40,45-47]; 12 scheduled or nonclinical [24,29,32,33,35,38,41,45,46]) compared to an on-demand coaching component (8 on demand or clinical [22,24,27,29,30,37-39]; 9 on demand or nonclinical [24,25,29,34,37,38,42,44,46]); 11 of the studies incorporated 2 or more kinds of coaching [18,20,21,25,29,33,34,38,44-46].

There was less variability as to who provided the coaching. Clinicians acted as coaches in 18 of the studies [22-31,36-41,45,47], nonclinicians in 3 studies [32,35,42], and peers in 3 studies [34,37,44]. Moreover, 4 studies did not specify who acted as coaches [33,34,44,46]. In addition, the majority of studies did not specify the type of training that the coaches completed for their role; 16 studies did not specify the type of training [22-25,27,30,32,33,35-45], 1 study specified that training was not conducted [34], and 7 studies specified that coaches underwent training of some kind [26,28,29,31,38,41,45]. Of the studies that did specify the training for the coaches, there was high variability; 1 study noted the coaches’ training was their standard training as a part of their clinical psychology program [29]; another noted the training was a “1-day workshop in using the self-help program and on how to write the weekly feedback, based on case material from earlier trials” [38], while another only stated their training was “based on the supportive accountability model” [41]. Only 1 study offered a protocol for the training offered [46].

Only 3 studies used the social coaching model [34,37,44]; 1 of these trials used only the social model [34], while 2 of them used the social model along with the coach model [37,44]. Boettcher et al [34], who used only the social model, examined the efficacy of a smartphone app called Challenger in reducing anxiety symptoms in individuals with social anxiety disorder. The participants were randomized to use Challenger and a self-help program simultaneously, the self-help program for 6 weeks followed by the Challenger app for 6 weeks, or a waitlist control. Challenger used cognitive behavioral therapy techniques to encourage its users to complete small exposure and behavioral challenges in everyday life. The skills gradually increased in difficulty. After each skill, the user is able to complete a reflection of the task, which is sent to another user who is able to respond with constructive feedback. The participants in Schlosser et al [37] and Roepke et al [44] were able to interact with other app users or use a forum and recruit social support from Facebook, respectively.

There was evidence of a dose effect in only 1 study. In particular, a pilot randomized controlled trial conducted by Pfeiffer et al [47], exploring psychotherapeutic text messaging for depression, found that change in behavioral activation was correlated with specifically 6 weeks of receiving acceptance and commitment therapy-based messages (ρ=-0.25; *P*<.05), as opposed to 12 weeks, at which point there was no correlation observed [47]. Studies used varying measures of engagement and efficacy of the respective smartphone technologies; 38.5% (n=10) reported percent completion of the program [21-23,28,29,32,34,35,40,46], 19.2% (n=5) reported app use per week or day [24,27,30,36,46], 19.2% (n=5 reported the retention or dropout rate [37,38,43,45,46], and 7.7% (n=2) reported the number of logins to the program [33,44]. However, only 10 studies reported the duration of time spent per coaching interaction [22-25,29-31,41,42,46], and many did not directly assess the influence of coaching on the results.

Finally, there was high variability in the mode of contact used across the studies; 13 (50%) of the studies used 2 or more means of contact [22,24,25,27,29,30,32,37,41,42,44-46]. Phone calls were most commonly used to contact participants (14, 53.8%) [22-24,27,29,30,32,33,35,37,41,42,45,46], followed by emails (7, 26.9%) [22,25,27,32,41,43,44], then in-person meetings (6, 23%) [26,28,31,36,42,45], in-app messaging (5, 19.2%) [24,29,30,34,37], text messaging (5, 19.2%) [25,27,38,46,47], FaceTime or teleconference (2, 7.7%) [37,39], app notifications (1, 3.8%) [39], and Facebook (1, 3.8%) [44].

**Table 1:** Summary of coding metrics.

Criteria and coding specifications		Number of studies, n (%)
**Type of training**		
	Specified	9 (35)
	Unspecified	16 (62)
**Mode of contact**		
	Email	7 (27)
	Phone call	14 (54)
	In-app message	5 (19)
	Text message	5 (19)
	In-person	6 (23)
	FaceTime or teleconference	2 (8)
	Notifications	1 (4)
	Facebook	1 (4)
**On demand vs scheduled**		
	On demand	12 (46)
	Scheduled	21 (81)
**Clinical vs nonclinical**		
	Clinical	19 (73)
	Nonclinical	14 (54)
**Clinician vs nonclinician vs peer**		
	Clinician	18 (69)
	Nonclinician	3 (12)
	Peer	3 (12)
	Not specified	4 (15)
**Time spent per interaction**		
	Specified	11 (42)
	Not specified	15 (58)
**Evidence of dose effect**		
	No	25 (96)
	Yes	1 (4)
**Social vs coach model**		
	Social	24 (92)
	Coach	5 (19)
**Participants compensated**		
	Yes	17 (65)
	No	9 (45)
**Participants received smartphone**		
	Yes	4 (15)
	Yes, if necessary	7 (27)
	No	16 (62)
**Remote study**		
	Yes	12 (46)
	No	14 (54)

## Discussion

Coaching offers a solution to engagement challenges with digital mental health, but its interpretation and implementation remain heterogeneous, consistent with our hypothesis. While our results are not a comprehensive review, they offer a selected sample across the mental health app literature, which highlights the diversity of efforts and results when applying different models of coaching to support apps. A lack of consensus around coaching protocols and outcomes precludes discussion of whether coaching may be a covariate, confounder, moderator, or mediator for clinical improvement with apps. While we were not able to explicitly measure the therapeutic alliance construct within this work, the heterogeneity found across coaching modalities may suggest a lack of consensus regarding how to best foster a digital therapeutic alliance [48] between the patient and clinicians. Recent studies not captured in our sample have employed the Digital Working Alliance Inventory to measure alliance with apps and suggested that such an alliance may predict app engagement [49], highlighting the significance of future research and standardization around this concept.

The high degree of heterogeneity reflected in our results suggests the versatility of coaching and its ability to easily adapt to unique circumstances. Coaching was feasible across all platforms ranging from text messages to social media and for both on-demand and scheduled interactions. Coaches were also able to support completely remote studies (defined as specifically involving no synchronous interactions) as well as offer face-to-face services in meeting with participants in other studies. While a clinician served the role in 69.2% of studies, the role is also accessible to other people including those with no formal training.

One challenge around understanding the efficacy of coaching, beyond the heterogeneity of the role and studies, is that training protocols, fidelity to those protocols, and coaching specific outcomes are often not reported. Without understanding how coaches are trained and if they adhere to that training during the study, it is impossible to understand what support is actually being delivered. Study metrics reports by coach instead of participant and cohort may also offer productive data toward understanding the impact of this role. While no studies measured outcomes such as the Working Alliance Inventory, alliance-specific measures would offer information into potential mechanisms of action.

However, the results from this paper offer several paths forward. These results suggest that clinical vs nonclinical staff can serve in coaching roles, and scheduled vs on-demand support can also both be feasible. Crowdsourcing peer support via social networks or small internal networks also appears feasible. As the role and best practices evolve, clinics can implement the methods that best match their local needs and resources. The different models presented in this paper can serve as examples in building new coaching services and provide measures to consider during implementation. While beyond the immediate scope of this article, protocols around digital mental health coaching are emerging and can serve as further reference [50,51]. Of note, neither of these protocols or earlier versions of them was used in any paper reviewed.

Our results are in line with prior works that have examined coaching around mental health apps. In a 2020 paper, Callejas et al [51] reported on selected examples and noted a need for more data around engagement and mechanisms of action underlying coaching. A lack of consensus around app engagement measures has also been found in recent reviews [52,53].

A chief limitation of this work is that it draws a sample from only 2 reviews of mental health app studies. Given that nearly every digital health study involves some degree of coaching (even if they are informal support from research assistants, which may not be reported), it is infeasible to conduct a broader review. Therefore, our goal was not to include every relevant paper, but rather to conduct a preliminary investigation into coaching techniques used by recent studies and identify trends. Other studies have specifically explored coaching and mental health apps. For example, in their 2019 paper, Mohr et al [54] found that coaching was associated with more downloads of a mental health app but not long-term engagement with that app. Our results are thus best interpreted as exploratory signals that suggest productive avenues for exploring coaching as well as guidance for understanding the high degree of heterogeneity that must be unpacked in new research efforts. The classification scheme used in this study was created de novo by our team given the state of this literature and can serve as a useful scaffold to create new versions in the future.

Coaching for mental health apps will continue to expand in scope, necessitating an understanding of its therapeutic potential and implementation into care settings. While current efforts around the role remain diverse, they suggest a flexibility necessary to support the evolving digital mental health space and to work across diverse populations and technologies.

## Appendix

Multimedia Appendix 1 Code book and coding results.

## Abbreviations

MOST+: enhanced moderated online social therapy

## References

[1] Bordin ES (1979). The generalizability of the psychoanalytic concept of the working alliance. Psychotherapy: Theory, Research & Practice.

[2] Ardito RB, Rabellino D (2011). Therapeutic alliance and outcome of psychotherapy: historical excursus, measurements, and prospects for research. Front Psychol.

[3] Carpenter-Song E, Acquilano SC, Noel V, Al-Abdulmunem M, Torous J, Drake RE (2022). Individualized Intervention to Support Mental Health Recovery Through Implementation of Digital Tools into Clinical Care: Feasibility Study. Community Ment Health J.

[4] Zhai Y (2021). A Call for Addressing Barriers to Telemedicine: Health Disparities during the COVID-19 Pandemic. Psychother Psychosom.

[5] Kim June-Ho, Desai Eesha, Cole Megan (2020). How The Rapid Shift To Telehealth Leaves Many Community Health Centers Behind During The COVID-19 Pandemic. Health Affairs Forefront.

[6] Byambasuren O, Beller E, Hoffmann T, Glasziou P (2020). mHealth App Prescription in Australian General Practice: Pre-Post Study. JMIR Mhealth Uhealth.

[7] Mordecai D, Histon T, Neuwirth E, Heisler WS, Kraft A, Bang Y, Franchino K, Taillac C, Nixon JP (2021). How Kaiser Permanente Created a Mental Health and Wellness Digital Ecosystem. NEJM Catalyst.

[8] Triana AJ, Gusdorf RE, Shah KP, Horst SN (2020). Technology Literacy as a Barrier to Telehealth During COVID-19. Telemed J E Health.

[9] Wisniewski H, Torous J (2020). Digital navigators to implement smartphone and digital tools in care. Acta Psychiatr Scand.

[10] Ben-Zeev D, Drake R, Marsch L (2015). Clinical technology specialists. BMJ.

[11] Noel VA, Carpenter-Song E, Acquilano SC, Torous J, Drake RE (2019). The technology specialist: a 21st century support role in clinical care. NPJ Digit Med.

[12] McEnery C, Lim MH, Knowles A, Rice S, Gleeson J, Howell S, Russon P, Miles C, D'Alfonso S, Alvarez-Jimenez Mario (2021). Social anxiety in young people with first-episode psychosis: Pilot study of the EMBRACE moderated online social intervention. Early Interv Psychiatry.

[13] Alvarez-Jimenez M, Rice S, D'Alfonso S, Leicester S, Bendall S, Pryor I, Russon P, McEnery C, Santesteban-Echarri O, Da Costa G, Gilbertson T, Valentine L, Solves L, Ratheesh A, McGorry PD, Gleeson J (2020). A Novel Multimodal Digital Service (Moderated Online Social Therapy+) for Help-Seeking Young People Experiencing Mental Ill-Health: Pilot Evaluation Within a National Youth E-Mental Health Service. J Med Internet Res.

[14] Tønning ML, Kessing LV, Bardram JE, Faurholt-Jepsen M (2019). Methodological Challenges in Randomized Controlled Trials on Smartphone-Based Treatment in Psychiatry: Systematic Review. J Med Internet Res.

[15] Tokgöz Pinar, Hrynyschyn R, Hafner J, Schönfeld Simone, Dockweiler C (2021). Digital Health Interventions in Prevention, Relapse, and Therapy of Mild and Moderate Depression: Scoping Review. JMIR Ment Health.

[16] Hur J, Kim B, Park D, Choi S (2018). A Scenario-Based Cognitive Behavioral Therapy Mobile App to Reduce Dysfunctional Beliefs in Individuals with Depression: A Randomized Controlled Trial. Telemed J E Health.

[17] Miner A, Kuhn E, Hoffman JE, Owen JE, Ruzek JI, Taylor CB (2016). Feasibility, acceptability, and potential efficacy of the PTSD Coach app: A pilot randomized controlled trial with community trauma survivors. Psychol Trauma.

[18] Kuhn E, Kanuri N, Hoffman JE, Garvert DW, Ruzek JI, Taylor CB (2017). A randomized controlled trial of a smartphone app for posttraumatic stress disorder symptoms. J Consult Clin Psychol.

[19] Lüdtke Thies, Pult LK, Schröder Johanna, Moritz S, Bücker Lara (2018). A randomized controlled trial on a smartphone self-help application (Be Good to Yourself) to reduce depressive symptoms. Psychiatry Res.

[20] Bakker D, Kazantzis N, Rickwood D, Rickard N (2018). A randomized controlled trial of three smartphone apps for enhancing public mental health. Behav Res Ther.

[21] Wahle F, Kowatsch T, Fleisch E, Rufer M, Weidt S (2016). Mobile Sensing and Support for People With Depression: A Pilot Trial in the Wild. JMIR Mhealth Uhealth.

[22] Watts S, Mackenzie A, Thomas C, Griskaitis A, Mewton L, Williams A, Andrews G (2013). CBT for depression: a pilot RCT comparing mobile phone vs. computer. BMC Psychiatry.

[23] Dagöö Jesper, Asplund RP, Bsenko HA, Hjerling S, Holmberg A, Westh S, Öberg Louise, Ljótsson Brjánn, Carlbring P, Furmark T, Andersson G (2014). Cognitive behavior therapy versus interpersonal psychotherapy for social anxiety disorder delivered via smartphone and computer: a randomized controlled trial. J Anxiety Disord.

[24] Gustafson DH, McTavish FM, Chih M, Atwood AK, Johnson RA, Boyle MG, Levy MS, Driscoll H, Chisholm SM, Dillenburg L, Isham A, Shah D (2014). A smartphone application to support recovery from alcoholism: a randomized clinical trial. JAMA Psychiatry.

[25] Ly KH, Trüschel Anna, Jarl L, Magnusson S, Windahl T, Johansson R, Carlbring P, Andersson G (2014). Behavioural activation versus mindfulness-based guided self-help treatment administered through a smartphone application: a randomised controlled trial. BMJ Open.

[26] Depp CA, Ceglowski J, Wang VC, Yaghouti F, Mausbach BT, Thompson WK, Granholm EL (2015). Augmenting psychoeducation with a mobile intervention for bipolar disorder: a randomized controlled trial. J Affect Disord.

[27] Faurholt-Jepsen M, Frost M, Ritz C, Christensen EM, Jacoby AS, Mikkelsen RL, Knorr U, Bardram JE, Vinberg M, Kessing LV (2015). Daily electronic self-monitoring in bipolar disorder using smartphones – the MONARCA I trial: a randomized, placebo-controlled, single-blind, parallel group trial. Psychol. Med.

[28] Ly KH, Topooco N, Cederlund H, Wallin A, Bergström Jan, Molander O, Carlbring P, Andersson G (2015). Smartphone-Supported versus Full Behavioural Activation for Depression: A Randomised Controlled Trial. PLoS One.

[29] Moëll B, Kollberg L, Nasri B, Lindefors N, Kaldo V (2015). Living SMART — A randomized controlled trial of a guided online course teaching adults with ADHD or sub-clinical ADHD to use smartphones to structure their everyday life. Internet Interventions.

[30] Ivanova E, Lindner P, Ly KH, Dahlin M, Vernmark K, Andersson G, Carlbring P (2016). Guided and unguided Acceptance and Commitment Therapy for social anxiety disorder and/or panic disorder provided via the Internet and a smartphone application: A randomized controlled trial. J Anxiety Disord.

[31] Hildebrandt T, Michaelides A, Mackinnon D, Greif R, DeBar L, Sysko R (2017). Randomized controlled trial comparing smartphone assisted versus traditional guided self-help for adults with binge eating. Int J Eat Disord.

[32] Mantani A, Kato T, Furukawa TA, Horikoshi M, Imai H, Hiroe T, Chino B, Funayama T, Yonemoto N, Zhou Q, Kawanishi N (2017). Smartphone Cognitive Behavioral Therapy as an Adjunct to Pharmacotherapy for Refractory Depression: Randomized Controlled Trial. J Med Internet Res.

[33] Ben-Zeev D, Brian RM, Jonathan G, Razzano L, Pashka N, Carpenter-Song E, Drake RE, Scherer EA (2018). Mobile Health (mHealth) Versus Clinic-Based Group Intervention for People With Serious Mental Illness: A Randomized Controlled Trial. Psychiatr Serv.

[34] Boettcher J, Magnusson K, Marklund A, Berglund E, Blomdahl R, Braun U, Delin L, Lundén C, Sjöblom K, Sommer D, von Weber K, Andersson G, Carlbring P (2018). Adding a smartphone app to internet-based self-help for social anxiety: A randomized controlled trial. Computers in Human Behavior.

[35] Bucci S, Barrowclough Christine, Ainsworth John, Machin Matthew, Morris Rohan, Berry Katherine, Emsley Richard, Lewis Shon, Edge Dawn, Buchan Iain, Haddock Gillian (2018). Actissist: Proof-of-Concept Trial of a Theory-Driven Digital Intervention for Psychosis. Schizophr Bull.

[36] Liang D, Han H, Du J, Zhao M, Hser Y (2018). A pilot study of a smartphone application supporting recovery from drug addiction. J Subst Abuse Treat.

[37] Schlosser D, Campellone Timothy R, Truong Brandy, Etter Kevin, Vergani Silvia, Komaiko Kiya, Vinogradov Sophia (2018). Efficacy of PRIME, a Mobile App Intervention Designed to Improve Motivation in Young People With Schizophrenia. Schizophr Bull.

[38] Stolz T, Schulz A, Krieger T, Vincent A, Urech A, Moser C, Westermann S, Berger T (2018). A mobile app for social anxiety disorder: A three-arm randomized controlled trial comparing mobile and PC-based guided self-help interventions. J Consult Clin Psychol.

[39] Faurholt-Jepsen M, Frost M, Christensen EM, Bardram JE, Vinberg M, Kessing LV (2019). The effect of smartphone-based monitoring on illness activity in bipolar disorder: the MONARCA II randomized controlled single-blinded trial. Psychol. Med.

[40] Krzystanek M, Borkowski M, Skałacka K, Krysta K (2019). A telemedicine platform to improve clinical parameters in paranoid schizophrenia patients: Results of a one-year randomized study. Schizophr Res.

[41] Stiles-Shields C, Montague E, Kwasny MJ, Mohr DC (2019). Behavioral and cognitive intervention strategies delivered via coached apps for depression: Pilot trial. Psychol Serv.

[42] Teng M, Hou Y, Chang S, Cheng H (2019). Home-delivered attention bias modification training via smartphone to improve attention control in sub-clinical generalized anxiety disorder: A randomized, controlled multi-session experiment. J Affect Disord.

[43] Enock PM, Hofmann SG, McNally RJ (2014). Attention Bias Modification Training Via Smartphone to Reduce Social Anxiety: A Randomized, Controlled Multi-Session Experiment. Cogn Ther Res.

[44] Roepke AM, Jaffee SR, Riffle OM, McGonigal J, Broome R, Maxwell B (2015). Randomized Controlled Trial of SuperBetter, a Smartphone-Based/Internet-Based Self-Help Tool to Reduce Depressive Symptoms. Games Health J.

[45] Possemato K, Kuhn E, Johnson E, Hoffman JE, Owen JE, Kanuri N, De Stefano L, Brooks E (2016). Using PTSD Coach in primary care with and without clinician support: a pilot randomized controlled trial. Gen Hosp Psychiatry.

[46] Mohr DC, Tomasino KN, Lattie EG, Palac HL, Kwasny MJ, Weingardt K, Karr CJ, Kaiser SM, Rossom RC, Bardsley LR, Caccamo L, Stiles-Shields C, Schueller SM (2017). IntelliCare: An Eclectic, Skills-Based App Suite for the Treatment of Depression and Anxiety. J Med Internet Res.

[47] Pfeiffer PN, Henry J, Ganoczy D, Piette JD (2016). Pilot study of psychotherapeutic text messaging for depression. J Telemed Telecare.

[48] Henson P, Wisniewski H, Hollis C, Keshavan M, Torous J (2019). Digital mental health apps and the therapeutic alliance: initial review. BJPsych Open.

[49] Goldberg SB, Baldwin SA, Riordan KM, Torous J, Dahl CJ, Davidson RJ, Hirshberg MJ (2021). Alliance With an Unguided Smartphone App: Validation of the Digital Working Alliance Inventory. Assessment.

[50] Wisniewski H, Gorrindo Tristan, Rauseo-Ricupero N, Hilty D, Torous J (2020). The Role of Digital Navigators in Promoting Clinical Care and Technology Integration into Practice. Digit Biomark.

[51] Callejas Z, Griol D, Benghazi K, Noguera M, Chollet G, Torres M, Esposito A (2002). Measuring and Fostering Engagement with Mental Health e-Coaches. Companion Publication of the 2020 International Conference on Multimodal Interaction.

[52] Bradway M, Gabarron E, Johansen M, Zanaboni P, Jardim P, Joakimsen R, Pape-Haugaard L, Årsand Eirik (2020). Methods and Measures Used to Evaluate Patient-Operated Mobile Health Interventions: Scoping Literature Review. JMIR Mhealth Uhealth.

[53] Ng MM, Firth J, Minen M, Torous J (2019). User Engagement in Mental Health Apps: A Review of Measurement, Reporting, and Validity. Psychiatr Serv.

[54] Mohr DC, Schueller SM, Tomasino KN, Kaiser SM, Alam N, Karr C, Vergara JL, Gray EL, Kwasny MJ, Lattie EG (2019). Comparison of the Effects of Coaching and Receipt of App Recommendations on Depression, Anxiety, and Engagement in the IntelliCare Platform: Factorial Randomized Controlled Trial. J Med Internet Res.

